# Psychotherapy Training on Psychological Mindedness in a Japanese Nurse Population: Effects and Personality Correlates

**DOI:** 10.3390/healthcare5030043

**Published:** 2017-08-08

**Authors:** Tomomi Saito, Satoru Takeda, Yukiko Yamagishi, Reiko Kubo, Toshinori Kitamura

**Affiliations:** 1 Kitamura Institute of Mental Health Tokyo, Tokyo 151-0063, Japan; tsaito2008@hotmail.co.jp (T.S.); yamagishiyukiko@gmail.com (Y.Y.); 2 Department of Obstetrics and Gynecology, School of Medicine, Juntendo University, Tokyo 113-0033, Japan; stakeda@juntendo.ac.jp; 3 Kumamoto Prefectural Government, 6-18-1, Suizenji, Chuo-ku, Kumamoto 862-8570, Japan; kubo-r@pref.kumamoto.lg.jp; 4 Department of Psychiatry, Graduate school of Medicine, Nagoya University, Nagoya 464-0814, Japan

**Keywords:** psychotherapy training, psychological mindedness, personality trait, Temperament and Character Inventory, self-directedness, harm-avoidance

## Abstract

***Aims and objective*:** The aim of this study was to determine whether the training would influence the psychological mindedness of nurses and midwives. In addition, we explored the relationship of the change of psychological mindedness before and after the training and the correlation with their personality traits. ***Background*:** It is important for perinatal health professionals such as nurses and midwives to acquire intervention skills such as psychotherapy and counselling techniques. We think that one of the essential requisites is psychological mindedness. ***Method*:** A total of 45 perinatal health professionals who participated in the postpartum depression prevention programme were distributed a set of questionnaires including the Psychological Mindedness Scale (PMS) and Temperament and Character Inventory (TCI) at the beginning and end of the training. ***Results*:** The PMS scores increased significantly after the training. A structured equation modelling suggested that PMS and self-directedness predicted each other whereas PMS predicted low harm avoidance. ***Conclusion*:** These findings indicate that the psychological mindedness of nurses and midwives could be advanced by a course of training and that this could be supported by high self-directedness. The harm avoidance trait may be reduced by increased psychological mindedness. ***Relevance to clinical practice*:** Nurses and nursing students are apt to psychological skill training in the advancement of psychological mindedness.

## 1. Introduction

Perinatal health professionals such as nurses and midwives are responsible for the physical as well as the psychological care for pregnant women and mothers of infants. This is because they have the advantage of easy access to pregnant women and are thus in a position to provide them with psychological support. Nevertheless, their graduate and on-the-job education traditionally places greater emphasis on somatic care. There should be a shift towards providing knowledge about perinatal mental illness and teaching intervention skills such as psychotherapy and counselling techniques [1].

We think that one of the essential requisites for nurses to provide psychological care is psychological mindedness. This is “a willingness to try to understand self and others, a belief in the benefits of discussing one’s problems, openness to new ideas, and access to one’s feelings” [2]. Highly psychologically minded people observe the relationships among thoughts, feelings, and behaviors in order to understand themselves and others [3]. Psychological mindedness is associated with an awareness of cognitive and affective processes, which are—in theory—of great interest to highly psychologically minded individuals. People high in psychological mindedness tend to be aware of self and others [4]. As suggested by previous works on psychological mindedness and attachment [5,6], psychological mindedness may develop in securely attached, well-adjusted individuals. The psychologically minded individuals’ interest and curiosity in psychological life might override the anxiety felt by less psychologically minded people in similar situations. Psychologically minded individuals employ causal and attributional models that are in line with psychology. They do so by believing in the benefits of discussing their problems, having access to their feelings, showing interest in understanding themselves and others, and professing an openness to change [7]. Psychological mindedness has been regarded as an important quality in psychotherapy patients and in psychotherapists [8,9]. For psychoanalysis especially, psychological mindedness is thought to be almost a prerequisite for both therapist and clients [10]. We believe that psychological mindedness would be important for not only patient selection but also therapist suitability for psychological intervention.

Personality traits may be associated with psychological mindedness. Thus, Beitel and colleagues’ [4] study suggested that a cognitive-style profile for psychologically minded individuals included a tolerance for ambiguity, a sense of personal agency, and a propensity for reality-oriented thinking. Each of these cognitive-style attributes is associated with psychological adjustment rather than pathology [7]. Beitel and Cecero [6] found that lower psychological mindedness was correlated with the neuroticism subscale of the NEO Five Factor Inventory [11]. It was positively associated with extraversion and openness to experiences [6]. Hence, it is necessary to further investigate the association of psychological mindedness with personality traits using methods other than the NEO Inventory.

Despite its clinical importance, it still remains to be studied whether the psychological mindedness of nurses can be improved through education. It is also unclear whether the improvement, if any, is related to a change of personality traits following skills training. We had an opportunity to educate nurses and nursing students as a part of the Japanese replication of the postpartum depression prevention programme [12].

A major depressive episode within three months after childbirth is an issue concerning not only the women themselves but also for the children they look after [13]. Pregnancy and the postpartum period can be framed as a dynamic role transition focusing on the parental role, adaptation to physical changes, and altered relationships with the spouse, other children, coworkers, or significant others [14]. Women must adapt to the physical and social changes brought about by pregnancy, adopt the new role of being a mother, and build new relationships. Moreover, an interpersonal dispute is “a situation in which the patient and at least one significant other person have nonreciprocal expectations about their relationship” [15]. During pregnancy and the postpartum period, women are likely to experience various emotional conflicts in themselves and social problems with others [16].

Interpersonal psychotherapy (IPT) has been reported as an effective treatment for postpartum depression [17]. Furthermore, prevention based on IPT has been advocated [18]. One such programme was developed by Zlotnick and colleagues [12]. They published two separate reports on the efficacy of their intervention method in preventing postpartum depression [12,17]. This was replicated in Japan [19].

The primary research question of the current study was whether the psychotherapy training adapted for the postpartum depression prevention programme would influence the psychological mindedness of participating nurses, midwives, and nursing students. The second aim of this study was to explore the relationship between the change of psychological mindedness before and after the training and the correlation with their personality traits.

## 2. Methods

### 2.1. Participants and Procedures

In 2007, we solicited midwives, community nurses, nurses, and nursing students attending a 5-session course of the psychological prevention programme of postnatal depression to participate in this study. After consenting to participate, the participants were administered a set of questionnaires twice: in the first and the last session of the 5-session training course. The participants included 25 midwives, 18 community health nurses, 2 nurses, and 1 nursing student. Their mean (SD) age was 42.0 (10.5) years old.

The participants attended five training sessions. The aim of this training course was to acquire preventive intervention skills for postnatal depression [12]. The training was composed of a series of lectures and role-playing sessions. Preventive intervention is based on the principles of interpersonal psychotherapy. It consists of four group sessions during pregnancy and a single group session after delivery. Each session lasts for about 60 min. The first session consisted of explaining the rationale for the program and psychoeducation on “baby blues” and postpartum depression. The second session focused on identifying role transitions to motherhood, changes associated with role transitions, and goals for successfully managing such situations. The third session was concerned with setting goals, developing support, and identifying potential interpersonal conflicts, especially once the baby was born. The fourth session taught skills for resolving interpersonal conflicts and reviewed the main themes of intervention. The booster session aimed to reinforce skills learned in the group sessions and to address any current or anticipated mood changes associated with interpersonal difficulties now that the newborn has arrived [12]. The goal of this preventive intervention is to increase women’s self-awareness regarding problems such as grief, disputes, and isolation, whether these occur during pregnancy or during the postpartum period. During this session, women learn how to successfully reduce excessive expectations and overcome maladaptive communication styles. They ultimately view their new role as more positive and are less reluctant to form new relationships [15].

We distributed a set of questionnaires to the participants just before the beginning of the first training session and at the end of the last training session.

### 2.2. Measurements

#### 2.2.1. Psychological Mindedness

We used the Psychological Mindedness Scale (PMS) [20]. The PMS is a 45-item self-report measure created to assess patient suitability for psychodynamic psychotherapy. The items are presented on a four-point scale and range from “strongly agree” (1) to “strongly disagree” (4). Higher scores indicate a greater psychological mindedness. Psychological mindedness is an individual’s ability to recognize and admit psychological and interpersonal problems, to see himself/herself in psychological terms, to use or to accept the use of psychological constructs, or to at least imagine the psychological causes of his/her symptoms and behavior. Cronbach’s alpha coefficients of the PMS score in this study were 0.84 and 0.93 for the pre- and post-training tests in this study, respectively (Table 1).

#### 2.2.2. Personality Traits

We used the Temperament and Character Inventory (TCI) [21]. This is a self-questionnaire developed to assess the seven dimensions of personality described by Cloninger and colleagues [21] with a total of 29 subscales. He revised the biosocial model of personality and posited seven domains of personality: four domains of temperament—Harm Avoidance (HA), Novelty Seeking (NS), Reward Dependence (RD), and Persistence (P)—and three domains of character—Self-Directedness (SD), Cooperativeness (CO), and Self-Transcendence (ST) [21]. SD and CO are thought of as reflections of personality maturation. The Japanese version [22] of the TCI is a 125-item short version instead of the 240-item full version. The Cronbach’s alpha coefficients of the TCI domain scores in this study were modest to substantial (Table 1).

### 2.3. Data Analyses

After calculating the means and SDs of all of the variables used in this study, we compared the scores of each variable before and after the training course. We then correlated them before and after the training course.

**Figure 1:**
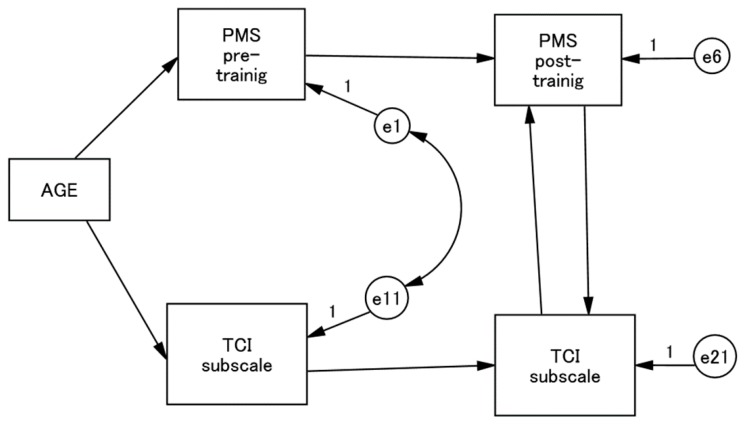
Path model of PMS and TCI subscale. Paths are all standardised. PMS, Psychological Mindedness Scale; TCI, Temperament and Character Inventory.

In order to examine the individual effects of each TCI subscale that showed a significant rise or fall after training on the increase of the PMS scores, we performed a series of non-recursive structured equation modelling (SEM) (Figure 1). Here the post-training PMS and TCI subscale scores were predicted by each of them rated at the pre-training examination. Whereas the error variables of PMS scores and each of the TCI subscale scores were correlated at the pre-training time point, we set bidirectional paths between the PMS and TCI subscale scores at the post-training time points. As an exogenous variable, we set the participants’ age that was designed to influence both the PMS and TCI subscale scores. The fit of each model with the data was examined in terms of chi-squared (CMIN), comparative fit index (CFI), and root mean square error of approximation (RMSEA). A good fit was indicated by CMIN/df < 2, CFI > 0.97, and RMSEA < 0.05, while an acceptable fit was indicated by CMIN/df < 3, CFI > 0.95, and RMSEA < 0.08 [23].

### 2.4. Ethical Consideration

This study was approved by the Institutional Review Board (IRB) of Kumamoto University School of Medical Sciences No. 269. Consent was obtained from the participants when distributing the questionnaire.

## 3. Results

As expected, the PMS scores were significantly higher after the training (*p* < 0.001: Table 2). So were the P, SD and ST scores. The HA scores decreased after the training (*p* < 0.001). At both pre- and post-training time points, PMS scores were correlated with higher CO and lower HA scores. They were also correlated with higher SD and ST at the post-training time point.

TCI subscales were correlated with each other. At both pre- and post-training time points, HA scores were negatively correlated with SD, CO, and ST. They were also negatively correlated with NS at the post-training time point. At both pre- and post-training time points, RD scores were correlated with higher CO. They were also correlated with higher SD at the post-training time point. At both pre- and post-training time points, P scores were correlated with higher ST. CO scores were correlated with higher P, SD, and ST at the pre-training time point.

**Figure 2:**
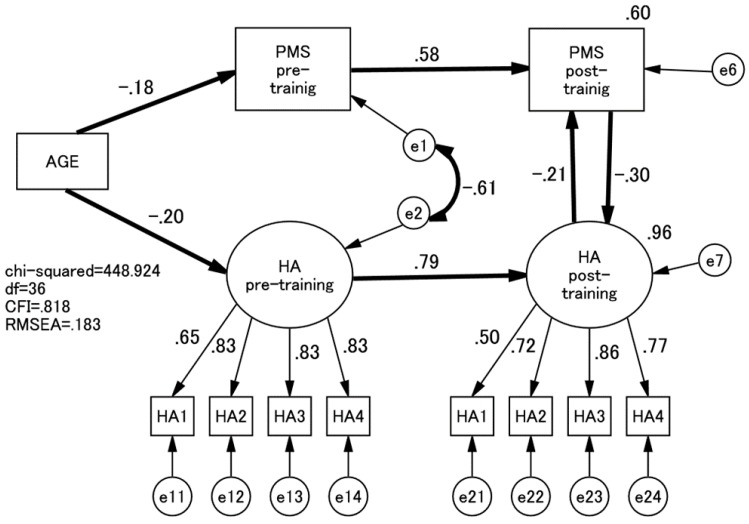
Path model of PMS and HA. Paths are all standardised. PMS, Psychological Mindedness Scale; HA, Harm Avoidance.

**Figure 3:**
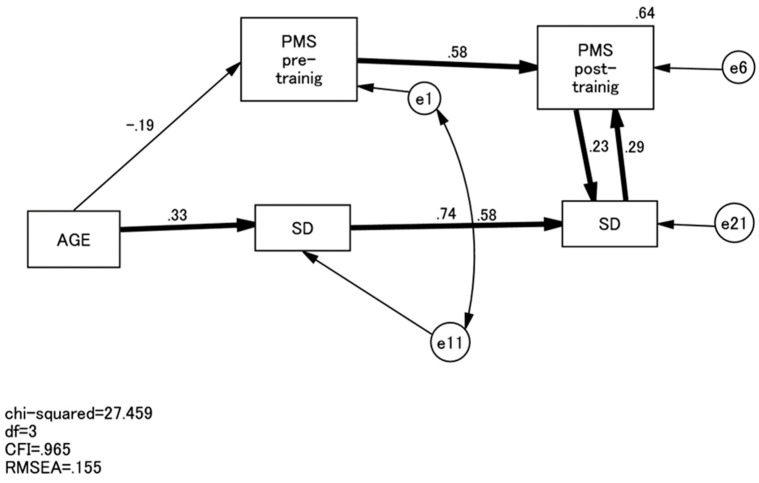
Path model of PMS and SD. Paths are all standardised. PMS, Psychological Mindedness Scale; SD, Self-directedness.

Three TCI subscales—HA, P, and SD—showed a statistically significant change after the whole training sessions. Therefore, we performed a series of non-recursive SEM to examine the effects of each of these three TCI subscales on the PMS scores after the training (Figure 1). After the training course, low HA scores significantly predicted PMS scores and vice versa (Figure 2). Similarly, high SD scores and PMS scores predicted each other after the training course (Figure 3). On the other hand, P scores and PMS scores failed to predict each other after training.

**Figure 4:**
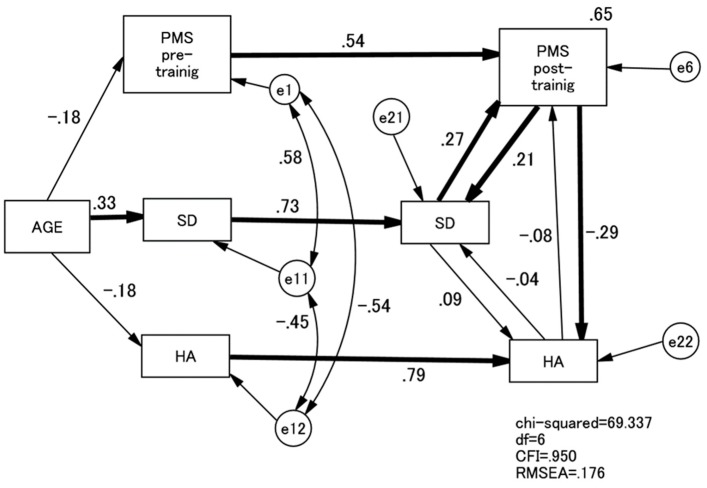
Path model of PMS, HA, and SD. Paths are all standardised. PMS, Psychological Mindedness Scale; SD, Self-directedness; HA, Harm Avoidance.

Hence, as a final model (Figure 4), we set up a non-recursive model in which PMS, SD, and HA were predicted by each other at the end of the training course. These three variables were also correlated with each other at the beginning of the training course. The final SEM showed an almost acceptable fit with the data (comparative fit index: CFI = 0.950, root mean square error of approximation: RMSEA = 0.176). At the end of the training course, the PMS scores were directly predicted by SD (β = 0.27, *p* < 0.001) scores that, in return, were predicted by the PMS scores (β = 0.21, *p* < 0.001). The post-training HA scores were inversely predicted by the PMS scores (β = −0.29, *p* < 0.001) but not vice versa. SD and HA after the training session did not predict each other.

## 4. Discussion

The psychological training for preventive intervention increased the PMS scores of the participants. This result indicates that psychological mindedness can improve over the postpartum depression prevention programme training course. This result is encouraging in that nurses and midwives may advance their skills based on their psychological mindedness. Although the present report is a secondary analysis of the data collected about 10 years ago, we do not think that the link between participants’ psychological mindedness, personality, and psychotherapy training will change substantially over the course of time.

This study also showed that SD and psychological mindedness influence each other. Nurses with high SD are more likely to improve in psychological mindedness after this training. Individuals who are self-directed and well-integrated are mature in personality, whereas those who are low in self-directedness are immature (i.e., developmentally delayed in character development), or have a personality disorder if they are adults [24,25]. Included in SD is the ability to have command of his/her own behavior to accommodate to the current situation so as to meet individually chosen goals and values [24]. In the process of nurturing this ability, one may come to possess the psychological facility to avoid the onset of depression. It may be that those high in SD are more capable of coping efficiently and successfully with difficult life situations [21]. SD is also related to the number of supportive people in the individual’s network [19]. Terry, Rawle, and Callan [26] showed that a better perception of social support by pregnant women could predict efficient coping behaviour after childbirth. Therefore, high SD may be related to both better social support and better coping behavior.

Cloninger [24] demonstrated that the development of well-being (i.e., presence of happiness and absence of sadness) depends on a combination of all three aspects of self-aware consciousness. He reported that there are some major stages of self-awareness along the path to well-being. The first stage of self-awareness is typical of most adults most of the time. Ordinary adult cognition involves a capacity to delay gratification in order to attain personal goals, but remains egocentric and defensive. The next stage of self-aware consciousness is typical of adults when they operate like a “good parents”. Good parents are allocentric in perspective—that is, they are capable of calmly considering the perspective and needs of their children and other people in a balanced way that leads to satisfaction and harmony. This state is experienced when a person is able to observe his own subconscious thoughts and consider the thought processes of others in a similar way to his observing his own thoughts. This ability is in line with the concept of psychological mindedness and it seems reasonable to conclude that psychological mindedness is directly predicted by SD.

HA is a trait that is characterized by low energy level. People high in HA are cautious, fearful, nervous, and passive [21]. They are shy in most social situations and easily feel tired. Hence, such a temperament is not suited for people who provide psychological support for patients. Not surprisingly, HA was strongly inversely related to psychological mindedness. It is of interest that HA was predicted by PMS scores at the end of the training sessions. That is, attendance at the present postpartum depression prevention programme course may reduce the level of HA. This is also encouraging that the participants of this training course may acquire skills as a therapist. 

Another issue derived from the present study is the role of the participants’ age. As shown in Figure 4 as well as Table 1, older age, though negatively associated with the PMS scores, predicted (significantly) higher SD and lower HA. This suggests that even though older nurses and midwives may be less psychologically minded at the start of the training course, they are more likely to acquire strong psychological mindedness over the course of the training via increased SD and lowered HA. Their personal experiences thus far may assist them in adapting themselves in psychological intervention training and contribute to the development of psychological mindedness.

The wellbeing of nurses may be a question of ability to manage with the situations in a satisfactory way. By studying psychological intervention skills, they may learn how to deal with issues of psychological problems of mothers presented in a clinical setting. This may lead to better self-efficacy and thus to better psychological mindedness. This aspect may also be an issue studied in future studies.

## 5. Limitations

Several limitations of this study should be noted. First, the present participants were not a random sample of nursing staff in Japan. They were strongly motivated in perinatal psychological care and voluntarily participated in the training course. Hence, they may be more apt to adopt psychologically minded attitudes after the present training. The results of this study cannot be generalized to nurses and nursing students without careful consideration.

Second, it still remains to be studied whether the increase of psychological mindedness will lead to actual skills in psychologically supportive intervention in clinical settings. The interpersonal psychotherapy changes practical aspects of the nurse’ and patients’ management. It is imperative to observe if and how much the education conducted in this study produces the real change of nurses’ skill of psychological intervention as well as change the patients’ mental state and ability to care of babies. The present study was a preliminary investigation such as a part an intervention study. When in a study of the effects of the psychological prevention of postnatal depression, we should pay particular attention to the nurses’ and mothers’ distress in terms of the nurses’ psychotherapeutic skills and psychological mindedness.

Third, although psychological intervention skills are very important for nurses and midwives, not all of them are suited for such clinical work. It may be of importance to identify those nurses and midwives who deserve a training course like ours. Our results suggest that the training is suited to those who are highly psychologically minded, high in SD and low in HA, and have had longer experiences in clinical settings. However, this is another important focus for future studies.

The changes of psychological mindedness and personality traits observed in this study should be interpreted with caution. They may be a product of a more efficient behavior and more clear aims and objectives of the respective roles perceived by the nurses. These should be further studied in future studies.

## 6. Conclusions

Taking these methodological drawbacks into consideration, our study indicates that nurses and nursing students are apt to psychological skill training in the advancement of psychological mindedness. This may also be influenced by personality traits, in particular, self-directedness. Endeavour to enhance nurses’ psychological mindedness may lead to the betterment of the work of studies performed by perinatal health professionals.

## Figures and Tables

**Table 1:** The internal consistency of all the variables used in the present study and their means and SD at the beginning and the end of training session course.

Variables	Cronbach		Mean (SD)		*t*
	Pre-Training	Post-Training	Pre-Training	Post-Training	
PMS	0.84	0.93	83.1 (10.1)	87.4 (14.1)	6.4 ***
HA	0.90	0.87	30.9 (8.4)	29.0 (7.3)	7.4 ***
NS	0.76	0.65	26.9 (5.6)	27.2 (4.5)	1.4
RD	0.75	0.69	31.2 (3.4)	31.1 (4.6)	0.5
P	0.46	0.39	15.9 (2.6)	16.7 (2.5)	5.1 ***
SD	0.85	0.79	43.9 (7.6)	45.4 (7.2)	5.4 ***
CO	0.82	0.89	50.9 (6.9)	51.3 (7.9)	1.2
ST	0.85	0.89	20.1 (6.2)	20.8 (7.0)	2.3 *

* *p* < 0.05; *** *p* < 0.001. PMS, Psychological Mindedness Scale; HA, Harm Avoidance; NS, Novelty Seeking; RD, Reward Dependence; P, Persistence; SD, Self-Directedness; CO, Cooperativeness; ST, Self-Transcendence.

**Table 2:** Correlations of all the variables used in the present study.

Varibles	PMS	HA	NS	RD	P	SD	CO	ST
PMS	-							
HA	−0.49 ** −60 **	-						
NS	0.08 0.14	−0.384 * −0.453 **	-					
RD	0.40 * 0.69 *	0.051 0.308	−0.015 −0.223	-				
P	0.36 * 0.28	−0.312 * −0.132	−0.081 −0.378 *	0.204 0.389 *	-			
SD	0.39 * 0.67 **	−0.455 ** −0.507 **	−0.187 0.062	0.024 0.442 **	0.165 0.125	-		
CO	0.73 ** 0.89 **	−0.394 ** −0.549 **	−0.179 −0.029	0.524 ** 0.715 **	0.525 ** 0.377 *	0.532 ** 0.730 *	-	
ST	0.26 0.45 **	−0.468 ** −0.419 **	0.057 0.033	0.237 0.365 *	0.558 ** 0.624 **	0.131 0.088	0.436 ** 0.396 *	-
age	−0.204 ** 0.062	−0.179 ** −0.096	−0.199 ** 0.062	−0.080 −0.088	−0.013 −0.164 **	0.359 *** 0.223 ***	0.078 0.082	0.053 −0.085

* *p* < 0.05; ** *p* < 0.01; *** *p* < 0.001. PMS, Psychological Mindedness Scale; HA, Harm Avoidance, NS, Novelty Seeking, RD, Reward Dependence; P, Persistence; SD, Self-directedness; CO, Cooperativeness; ST, Self-transcendence. The upper and lower figures represent correlations at the beginning and the end of the training sessions, respectively.
